# Epicardial adipose tissue around the superior vena cava: A single center study of factors related to atrial fibrillation

**DOI:** 10.7555/JBR.36.20220047

**Published:** 2022-06-20

**Authors:** Xingxing Sun, Jun Wang, Qing Yan, Weizhu Ju, Fengxiang Zhang, Gang Yang, Kai Gu, Hailei Liu, Zidun wang, Xiaohong Jiang, Mingfang Li, Di Xu, Yi Xu, Hongwu Chen, Minglong Chen

**Affiliations:** Department of Cardiology, the First Affiliated Hospital of Nanjing Medical University, Nanjing, Jiangsu 210029, China

**Keywords:** atrial fibrillation, superior vena cava, epicardial adipose tissue, low voltage area

## Abstract

The superior vena cava (SVC) is the main component of non-pulmonary vein (PV) ectopy in patients with atrial fibrillation (AF). Researchers have found that epicardial adipose tissue (EAT) volume is related to the AF substrate, which can be defined by the low voltage area (LVA). This study aimed to investigate the relationship between SVC-EAT and SVC-AF. Twenty-six patients with SVC-AF triggers were identified as the SVC-AF group. Other three groups were defined and included as the LVA-AF group (LVA>5%), non-LVA-AF group (LVA<5%), and physical examination (PE) group. EAT around left atrium (LA-EAT) and SVC-EAT volumes were obtained using a cardiac risk assessment module. According to the SVC/LA-EAT ratio, there are significant differences between the SVC-AF group and the three control groups (the SVC-AF group 0.092±0.041 *vs.* the LVA-AF group 0.054±0.026, the non-LVA-AF group 0.052±0.022, and the PE group 0.052±0.019, all *P*<0.001). Receiver operating characteristic curve analysis suggests the optimal cut-off point of SVC/LA-EAT ratio is 6.8% for detecting SVC-AF patients, with 81.1% sensitivity, 73.1% specificity, and an area under the curve of 0.83 (95% confidence interval, 0.75–0.91). Those with SVC-AF have a higher SVC/LA-EAT ratio and empirical SVC isolation could be considered if the SVC/LA-EAT ratio was over 6.8%.

## Introduction

Pulmonary vein antrum isolation (PVAI) is a standard approach to treating atrial fibrillation (AF) in patients who are resistant to antiarrhythmic interventions ^[ 1– 2] ^. However, researchers have also found that up to 11% of patients also encounter non-pulmonary vein (non-PV) triggers. The superior vena cava (SVC) is the main component of non-PV ectopy in patients with AF, with prevalence of approximately 1.6% of paroxysmal AF (PAF) patients and 2.0% of persistent AF patients ^[ 3] ^. It has also been shown that empirical PVAI plus SVC isolation does not reduce overall AF recurrence. Although, the PVAI plus SVC isolation strategy can be effective if AF originates from the SVC ^[ 4] ^. A previous study found that SVC-AF had a longer SVC sleeve during the procedure ^[ 5] ^. However, the best method before the procedure to predict SVC-AF has yet to be determined.

Epicardial adipose tissue (EAT) is a unique visceral adipose tissue located in close proximity to the myocardium and coronary arteries, playing its unique functions. Studies found that moderate EAT can protect the heart; however, excessive EAT can cause inflammation and put extra strain on the cardiovascular system, such as atrial fibrosis. It has been suggested that EAT is related to the AF substrate, which can be determined using low voltage area (LVA) analysis during the ablation procedure; moreover, the volume of EAT, detected by cardiac computerized tomography (CT) scan, positively correlates with AF development ^[ 6– 10] ^.

So, we hypothesize that high burden of EAT surrounding the SVC (SVC-EAT) might correlate with SVC-AF in patient with SVC trigger. In this study, we obtained EAT measurements from around the SVC and left atrium (LA) using CT and attempted to drive a method that would predict SVC-AF before the procedure, by analyzing the correlation between SVC-EAT and SVC-AF.

## Subjects and methods

### Study population

From January 2017 to May 2021, 26 patients with AF, confirmed as the source of SVC during the radiofrequency ablation procedure, were enrolled in the SVC-AF group. All patients had been examined with cardiac CT, before excluding those with thrombi.

Forty-nine patients with a non-SVC-AF source were categorized as LVA-AF ( *i.e.*, LVA>5%) or the non-LVA-AF group (LVA<5%), according to voltage mapping. Twenty-five people without AF who underwent routine physical examinations were included in the PE group. All controls were matched with participants in the SVC-AF group according to biological sex and age. A flow chart of this study design has been provided in ***Supplementary Fig. 1*** (available online).

Patients aged >18 years and <80 years were eligible for catheter ablation if they had new onset or under-treated paroxysmal, persistent, or long-standing persistent AF, according to the consensus statement ^[ 11] ^. Exclusion criteria stipulated that left ventricular ejection fraction (LVEF) <35%, those with one or more co-morbidities, or severe valvular heart disease.

All patients provided written informed consent before participating, and this study was approved by the Human Research Ethics Committee of the First Affiliated Hospital of Nanjing Medical University (approval No.: 2015-SR-085).

### Cardiac CT protocols and image acquisition

Images were acquired using CT technologies; SOMATOM Force and SOMATOM Definition (Siemens Healthineers, Germany) with an ECG-gated dual-source single-energy protocol covering the entire heart. Acquisition parameters were as follows: retrospective ECG-gated protocol; automatic tube voltage adjustment (Care kV; Siemens) from 80 to 120 kVp; automatic tube current modulation ranging between 150 and 400 mAs ^[ 6] ^. Beta-blockers and nitroglycerine were used if necessary.

Images were obtained following the administration of a contrast medium *i.e.*, 370 mg iodine/mL (Ultravist, Bayer Schering Pharma, Germany) *via* a 20-G intravenous catheter, followed by 40 mL saline flush using a dual-head power injector. Injection duration was determined by scan time plus eight seconds and the flow rate ranging from 4.0 to 6.0 mL/s was set according to body weight, heart rate, and tube voltage. Delayed images (those taken 30 seconds after the first phase) were obtained to rule out left atrium appendage (LAA) thrombus, and the relevant methods are consistent with other studies of our center ^[ 12] ^.

### Epicardial adipose tissue measurements

**Figure 1:**
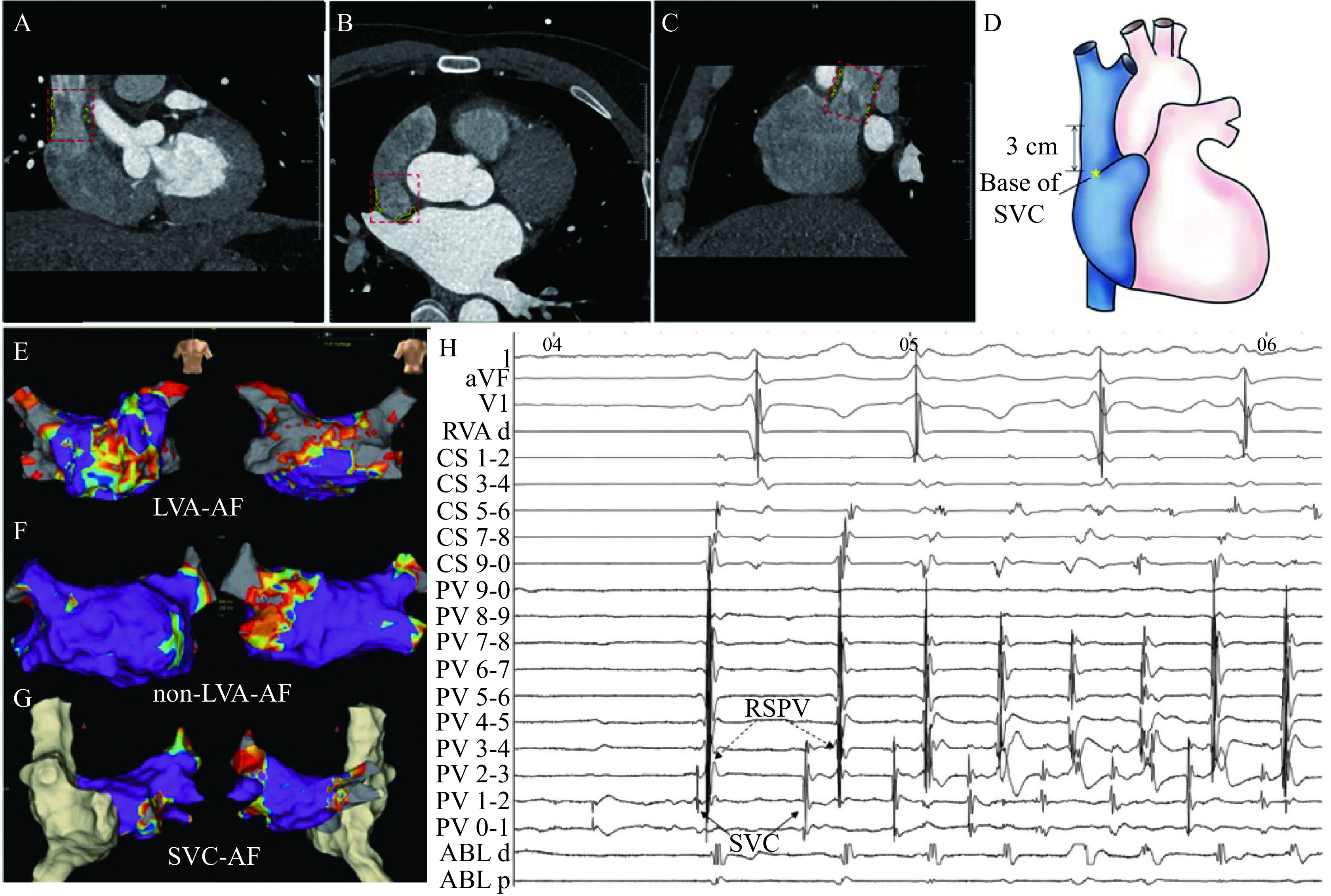
Schematic representation of the semiautomated SVC-EAT measurement method, LVA voltage percentage of different groups, and SVC triggered AF.

Post-processing was performed with a dedicated prototypical Syngo Frontier software (Siemens Healthineers) using a Cardiac Risk Assessment module to obtain the LA-EAT volume and SVC-EAT volume. Posterior LA EAT was manually traced from the base of the LA until the mitral annulus. SVC-EAT was manually traced from the junction of the right atrial appendage and SVC to 3 cm above the base of the SVC ( ***Fig. 1A*** **–** ***D*** and ***Supplementary Fig. 2 ***).

Cross-sectional areas, our regions of interest (ROI) of the SVC base, and 1 cm, 2 cm, and 3 cm above the SVC base, were measured manually. EAT volumes were obtained using a semi-automated method, and fat was recognized using threshold attenuation values of −50 to −150 HU in axial slices from the bifurcation juncture in the pulmonary trunk to the diaphragm.

CT data were assessed by two senior radiologists specializing in cardiovascular imaging with four years of experience in CT technologies. The average from the two measurements was accepted. If their measurements differed greatly, a more senior radiologist provided the final decision.

### Ablation and voltage mapping procedure

Ablation and voltage mapping procedures were similar to a related, previously published study ^[ 13] ^. In short, PVAI was firstly performed, if the AF was still present or sustainable, then cardioversion was delivered until AF convert to sinus rhythm, voltage mapping was performed using high-density mapping by 3-D anatomical system.

Local voltage was defined as the amplitude of local bipolar electrogram from positive peak deflection to negative peak deflection. LVA was defined as an area with bipolar peak-to-peak voltage amplitudes <0.5 mV and covering >5% of the LA surface area ^[ 9] ^, which was defined as the LA body area without the PV antrum regions, LAA orifice, and mitral valve. Scar areas were determined as areas with bipolar peak-to-peak voltage amplitudes <0.1 mV.

### SVC-AF definition and SVC isolation

AF originated from SVC was identified in the SVC-AF group during procedures. SVC trigger definitions were established using the following criteria: premature atrial contraction trigger AF was recorded by multi-polar catheter (St. Jude Medical, USA) in SVC, as mentioned in ***Fig. 1H***. The ablation strategy for SVC triggers was SVC isolation. SVC isolation was obtained by ablating approximately 1 cm above the SVC-RA junction with the guidance of 3D electro-anatomical mapping and a circular mapping catheter placed near the SVC-RA junction ^[ 14] ^. Before the ablation procedure, pacing from ABL-catheter was provided to identify the phrenic nerve area. This provided no (or at least, less) power to avoid injuring the phrenic nerve. Phrenic nerves were captured through pacing within SVC after isolation.

### Statistical analysis

Categorical variables are presented as frequencies and percentages. Continuous variables are presented as means with standard deviations (SD). These were compared using a standard one-way ANOVA if data were normally distributed. In the case of continuous data without a normal distribution, the Mann-Whitney-Wilcoxon test was used. Dunnett's multiple comparison test was used for intragroup comparisons. Categorical variables were assessed using a standard chi-square test or Fisher's exact test, where necessary. The threshold for statistical significance was set at P-value<0.05. Statistical analyses were conducted using SPSS (version 25.0).

## Results

### Patient characteristics

Baseline characteristics of the study population ( *N*=100, mean age 60.2 years) are summarized in ***Table 1***. Body surface area (BSA) for participants with AF were significantly greater compared to those without AF ( *P*=0.017). In all AF participants, the LVA-AF group had significantly less paroxysmal AF than the SVC-AF group and non-LVA-AF group. In addition, there were significantly higher NT-pro BNP and urea levels in the LVA group and significantly higher presentations of left atrial diameter in the LVA group than in the SVC-AF group and non-LVA group.

**Table 1:** Baseline characteristics of the study population

Characteristics	SVC-AF ( *N*=26)	LVA-AF ( *N*=24)	non-LVA-AF ( *N*=25)	PE ( *N*=25)	*P*-value
Female, *n* (%)	11 (42.3)	10 (41.7)	11 (44.0)	11 (44.0)	0.998
Age (years)	60.19±9.40	60.46±8.62	60.16±10.81	60.04±10.81	0.999
BMI (kg/m²)	25.27±3.10	25.79±2.74	24.97±3.40	23.48±3.52	0.076
BSA (m²)	1.77±0.15	1.81±0.19	1.75±0.20	1.65±0.18	0.017
Paroxysmal-AF, *n* (%)	21 (80.8)	8 (33.3)	19 (76.0)	—	0.001
Hypertension, *n* (%)	14 (53.8)	14 (58.3)	13 (52.0)	—	0.987
Diabetes mellitus, *n* (%)	5 (19.2)	3 (12.5)	4 (16.0)	—	0.813
CHA2DS2-VASc Score	1.69±1.57	1.79±1.10	2.04±1.95	—	0.763
NT-proBNP (ng/L)	297±318	1160±1196	617±899	—	0.002
TC (mmol/L)	4.41±1.03	4.16±0.90	3.97±0.94	—	0.404
TG (mmol/L)	1.51±0.71	1.34±0.48	1.60±1.58	—	0.565
HDL-C (mmol/L)	1.08±0.26	1.06±0.17	1.09±0.19	—	0.739
LDL-C (mmol/L)	2.75±0.71	2.54±0.74	2.30±0.69	—	0.145
Urea (mmol/L)	5.16±1.08	6.38±1.61	5.75±0.97	—	0.008
Cr (μmol/L)	70.00±17.39	76.88±15.00	69.31±17.65	—	0.152
UA (μmol/L)	342±91	387±132	335±107	—	0.210
FT3 (pmol/L)	4.56±0.57	4.58±0.62	4.54±0.75	—	0.768
FT4 (pmol/L)	17.07±3.01	16.45±1.88	17.02±2.33	—	0.615
TSH (mIU/L)	3.08±1.65	3.20±1.99	3.88±4.30	—	0.980
LVEF (%)	63.59±2.21	62.22±5.10	61.91±7.41	—	0.930
LAD (mm)	37.12±4.42	41.88±3.88	39.00±4.59	—	0.001
RAD (mm)	33.88±4.69	37.63±4.69	34.22±4.69	—	0.011
Values are presented as mean±standard deviation or *n* (%). SVC: superior vena cava; AF: atrial fibrillation; LVA: low voltage area; LA: left atrium; PE: routine physiological examination group; BMI: body mass index; BSA: body surface area; TC: Serum total cholesterol; TG: triglyceride; HDL-C: high-density lipoprotein cholesterol; LDL-C: low-density lipoprotein cholesterol; Cr: creatinine; UA: urea acid; FT3: Free Triiodothyronine; FT4: free thyroxine; TSH: Thyroid-stimulating Hormone; LVEF: left ventricular ejection fraction; LAD: left atrial diameter; RAD: right atrial diameter.					

***Fig. 1E**– **G*** show voltage maps with different percentages of LVA in the LVA-AF group, non-LVA-AF group, and SVC-AF group, respectively. ***Fig. 1H*** shows ectopic beats originating from SVC (solid black arrow) -triggered AF before isolation. It should be noted that the circular mapping catheter was positioned in the right superior PV (dotted black arrow), and the SVC far-field potential was recorded from the circular mapping catheter.

### Imaging variables of epicardial adipose tissue

**Figure 2:**
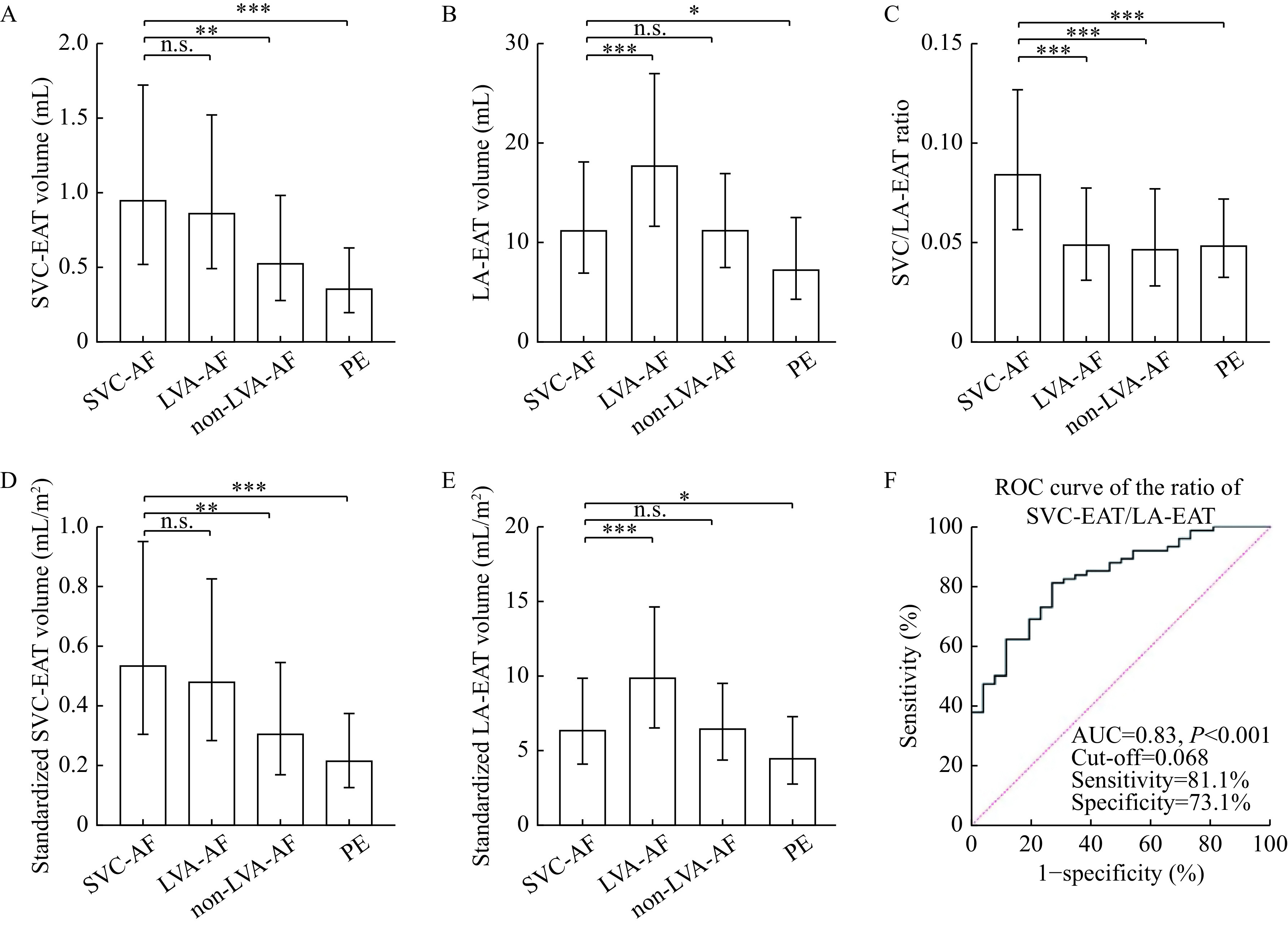
Imaging variables of each group.

Imaging variables within the four groups are provided in ***Table 2***. There was no significant difference in SVC-EAT between the SVC-AF group and LVA-AF group (1.13±0.71 *vs.* 1.02±0.67, *P*=0.84), but a significant difference between the SVC-AF group and the non-LVA-AF group (1.13±0.71 *vs.* 0.62±0.34, *p*=0.003) and the PE group (0.41±0.22, *P*<0.001), as shown in ***Fig. 2A***. The difference was statistically significant in LA-EAT between the SVC-AF group and the LVA-AF group (12.39±5.41 *vs.* 19.07±7.35, *P*<0.001, ***Fig. 2B***).

**Table 2:** Imaging variables of epicardial adipose tissue within the four groups

Variables	SVC-AF ( *N*=26)	LVA-AF ( *N*=24)	non-LVA-AF ( *N*=25)	PE ( *N*=25)	*P*-value
LA-EAT volume (mL)	12.39±5.41	19.07±7.35	12.13±4.93	8.27±3.90	<0.001
Standardized LA-EAT (mL/m ^2^)	6.91±2.69	10.53±3.87	6.91±2.65	4.96±2.18	<0.001
SVC-EAT volume (mL)	1.26±0.71	1.02±0.67	0.62±0.34	0.41±0.22	<0.001
Standardized SVC-EAT (mL/m ^2^)	0.63±0.37	0.56±0.34	0.35±0.18	0.25±0.12	<0.001
SVC/LA-EAT (%)	9.19±0.41	5.42±2.58	5.17±2.20	5.42±1.79	<0.001
SVC-0 area (cm)	3.29±1.24	4.06±1.62	3.17±1.37	2.34±0.91	0.001
SVC-1 area (cm)	2.73±1.01	3.24±1.30	2.70±1.02	1.90±0.63	<0.001
SVC-2 area (cm)	2.82±0.98	3.07±1.11	2.83±0.90	2.10±0.61	0.003
SVC-3 area (cm)	2.98±1.03	3.22±1.16	3.19±0.84	2.34±0.61	0.003
Values are presented as mean±standard deviation. LA: left atrium; SVC: superior vena cava; EAT: epicardial adipose tissue; AF: atrial fibrillation; LVA: low voltage area; PE: routine physiological examination group; standardized LA-EAT: LA-EAT divided by body surface area (BSA); standardized SVC-EAT: SVC-EAT divided by BSA. SVC-0, -1, -2, and -3 area: the cross-sectional areas of the base of SVC, 1 cm, 2 cm, and 3 cm above the base of SVC.					

The results were still similar after BSA standardization ( ***Fig. 2D*** and ***E***), as well as SVC-EAT standardized by ROI and LA-EAT standardized by LA volume. Although, the difference of LA-EAT/LA volumes in each group was not so significant ( *Supplementary Fig. 3A* and ***B ***, available online).

### Ratio of SVC/LA-EAT

A significant difference was also observed in the SVC/LA-EAT ratio between the SVC-AF group and the three control groups (SVC-AF group 0.092±0.041 *vs.* the LVA-AF group 0.054±0.026, non-LVA-AF group 0.052±0.022, and PE group 0.052±0.019, *P*<0.001, ***Fig. 2C***). As shown in ***Fig. 2F***, ROC curve analysis suggests the optimal cut-off point is 6.8% for detecting SVC-AF patients, with 81.1% sensitivity, 73.1% specificity, and an area under the curve (AUC) of 0.83 (95% confidenceinterval [CI], 0.75–0.91).

### Cross-sectional area of superior venacava

Up to the cross-sectional area of the SVC base, and 1 cm, 2 cm, and 3 cm above SVC ostium, a significant difference was obtained between the four groups, including the PE group, however, the difference was not significant between the AF groups defined here.

### Adverse events

There were no major complications observed in any of the patients.

## Discussion

The major findings in this study are as follows: 1) SVC-EAT volume in the SVC-AF group was significantly higher than that observed in the non-LVA-AF and PE group; 2) the SVC-AF group had the highest SVC/LA-EAT ratio among the four groups; 3) empirical SVC isolation may be considered if the SVC/LA-EAT ratio was over 6.8%.

EAT is a unique energy depot and serves as a biologically active organ, with considerable endocrine and inflammatory functions. There is no fascial layer separating the EAT and the myocardium, which allows for direct paracrine and vasocrine effects on the myocardium ^[ 15] ^. Accumulating evidence suggests that EAT is associated with AF initiation, perpetuation, and recurrence ^[ 16] ^. Quantitative and qualitative assessments of EAT using cardiac CT are of growing interest ^[ 17– 19] ^. Previous studies suggest that LA-EAT in AF patients is higher than that in patients without AF ^[ 20– 21] ^, and our study also has the same finding. Studies have shown that EAT positively correlates with the range of LVA ^[ 6– 10] ^. Our study found that the LA-EAT and SVC-EAT volumes of the LVA-AF group were both greater than that observed in the non-LVA-AF group, and the LA-EAT and SVC-EAT volumes in the AF groups were both significantly more than that in the PE group.

Studies have found that in non-PV areas ectopic foci exist in approximately 10% to 20% of AF patients. We also now know that the SVC is the main component involved in non-PV triggers by harboring 26% to 30% of non-PV foci ^[ 4, 22] ^. A previous study also found that it is necessary to isolate SVC, if AF is identified as originating in the SVC ^[ 4] ^. Empirical SVC isolation was strongly recommended if there was a long SVC sleeve or large SVC potential during the procedure ^[ 5] ^. Nevertheless, there is no predictive index before the ablation procedure because the SVC sleeve could not be detected by radiological technology. A previous study suggested that dilatation of the SVC is related to arrhythmogenic responses, and that the recurrence rate of AF was significantly higher in patients with a large SVC cross-sectional area ^[ 23] ^.

However, our study did not find a significant difference in the cross-sectional area of the SVC between the SVC-AF group and the non-SVC-AF group. The main reason for this might be that AF can spontaneously onset and triggered AF was identified to originate from the SVC in our study. Although, all patients in a previous study with repetitive atrial responses or non-sustained or sustained atrial tachyarrhythmia, were enrolled for analysis ^[ 23] ^. Additionally, the measurement method was different, and the SVC area was measured at the LA roof level in the previous study. This might have had a different SVC level because of the different LA sizes. Although, the cross-sectional area was measured in our study according to the anatomical landmark, the junction of the SVC and RAA and over 3 cm of the basal SVC.

We also observed a significant difference in SVC-EAT volume between the SVC-AF group and the non-LVA-AF group. While, there was no significant difference in SVC-EAT between the SVC-AF group and the LVA-AF group, which might be due to the general increase in EAT in the LVA-AF group. In a recently submitted study from our team, researchers defined the potential role of EAT in an LVA-AF group. That is, an increased EAT volume, especially EAT around the left atrium, is associated with the presence of LVA in patients with AF, and the percentage of LA-EAT/total EAT volume had the potential value to predict the severity of LVA in AF patients.

There was also a significant difference in the SVC/LA-EAT ratio in the SVC-EAT group compared with the remaining three groups. Furthermore, ROC curve analysis demonstrated an optimal cut-off point of 6.8% for detecting SVC-AF patients, with 81.1% sensitivity, 73.1% specificity, and the AUC was 0.83 (95% CI: 0.75–0.91). This is the first study to predict the SVC-AF origin before the procedure using the marker of the SVC/LA-EAT ratio, and SVC isolation may be considered if the SVC/LA-EAT ratio was over 6.8%. This study suggests that the EAT volume of the LVA-AF group was greater than that of the non-LVA-AF group. Additionally, there was no significant difference in the SVC/LA-EAT ratio among the LVA-AF, non-LVA-AF, and PE groups.

### Limitations

This was a single-center, non-randomized observational study and patients were not consecutively recruited because some patients did not receive cardiac CT scans. Additionally, there was a higher proportion of PAF in the SVC-AF and non-LVA-AF groups, but only 33% of patients had PAF in the LVA-AF group, which might have affected SVC-EAT and LA-EAT. CAD is a common comorbidity with persistent AF. In this study, the number of CAD/persistent AF in each group was 1/5 in the SVC-AF group, 5/16 in the LVA-AF group and 2/6 in the non-LVA-AF group. The differences in CHA _2_DS _2_-VASc Scores among the three AF groups were non-significant and the SVC/LA-EAT ratio was used for analysis. Therefore, the influence might only be slight. SVC-AF was identified in seven patients which might not rule out the impact of the first operation on the results. This might also have affected SVC-EAT and LA-EAT measures. Despite these limitations, it is useful to predict SVC-AF using a non-invasive method before ablation procedures, because some cases of SVC-AF are not triggered during the first procedure.

### Conclusions

The SVC-AF group had a high ratio of SVC-EAT divided by LA-EAT, and empirical SVC isolation may be considered if the SVC/LA-EAT ratio was over 6.8%. The relationship between EAT and SVC-AF needs to be validated in studies with larger samples, and the method of EAT measurement needs to be more simplified to be generalized in clinical work.
